# External validation of the Epic sepsis predictive model in 2 county emergency departments

**DOI:** 10.1093/jamiaopen/ooae133

**Published:** 2024-11-13

**Authors:** Daniel G Ostermayer, Benjamin Braunheim, Amit M Mehta, Jeremy Ward, Sara Andrabi, Anwar Mohammad Sirajuddin

**Affiliations:** Department of Emergency Medicine, McGovern Medical School, UT Health at the University of Texas Health Science Center at Houston, Houston, TX 77030, United States; Department of Health Informatics and Data Science, Harris Health System, Houston, TX 77401, United States; Department of Emergency Medicine, McGovern Medical School, UT Health at the University of Texas Health Science Center at Houston, Houston, TX 77030, United States; Department of Surgery, Baylor College of Medicine, Houston, TX 77030, United States; Department of Emergency Medicine, Baylor College of Medicine, Houston, TX 77030, United States; Department of Health Informatics and Data Science, Harris Health System, Houston, TX 77401, United States

## Abstract

**Objective:**

To describe the diagnostic characteristics of the proprietary Epic sepsis predictive model best practice advisory (BPA) alert for physicians in the emergency department (ED).

**Materials and Methods:**

The Epic Sepsis Predictive Model v1.0 (ESPMv1), a proprietary algorithm, is intended to improve provider alerting of patients with a likelihood of developing sepsis. This retrospective cohort study conducted at 2 county EDs from January 1, 2023 to December 31, 2023 evaluated the predictive characteristics of the ESPMv1 for 145 885 encounters. Sepsis was defined according to the Sepsis-3 definition with the onset of sepsis defined as an increase in 2 points on the Sequential Organ Function Assessment (SOFA) score in patients with the ordering of at least one blood culture and antibiotic. Alerting occurred at an Epic recommended model threshold of 6.

**Results:**

The ESPMv1 BPA alert was present in 7183 (4.9%) encounters of which 2253 had sepsis, and not present in 138 702 encounters of which 3180 had sepsis. Within a 6-hour time window for sepsis, the ESPMv1 had a sensitivity of 14.7%, specificity of 95.3%, positive predictive value of 7.6%, and negative predictive value of 97.7%. Providers were alerted with a median lead time of 0 minutes (80% CI, −6 hours and 42 minutes to 12 hours and 0 minutes).

**Discussion:**

In our population, the ESPMv1 alerted providers with a median lead time of 0 minutes (80% CI, −6 hours and 42 minutes to 12 hours and 0 minutes) and only alerted providers in half of the cases prior to sepsis occurrence. This suggests that the ESPMv1 alert is adding little assistance to physicians identifying sepsis. With clinicians treating sepsis 50% of the time without an alert, pop-ups can only marginally assist in disease identification.

**Conclusions:**

The ESPMv1 provides suboptimal diagnostic characteristics for undifferentiated patients in a county ED.

## Background

Rapid and early detection and treatment of sepsis has been associated with a significant mortality benefit in hospitalized patients. Early notification of potentially septic patients may help improve the speed of treatment.^1^ Multiple Electronic Health Record (EHR)-based models and alerting systems for sepsis have been developed and evaluated across many clinical settings.^2–8^ Specific to the emergency department (ED), EHR-based alerts have shown sensitivities ranging from 10% to 100% and positive predictive values (PPVs) from 5.8% to 54%.^8^

The Epic Sepsis Predictive Model (ESPMv1), a penalized logistic regression, utilizes multiple variables such as demographic, radiology, laboratory, and medication data to predict the likelihood of sepsis.^9^ Many EDs use Epic (Epic Systems Corporation, Madison WI) for their EHR provider and many utilize one of many automated alerting systems for sepsis identification.^10^ ESPMv1, a proprietary algorithm, is intended to improve alerting of providers for patients with a likelihood of developing sepsis. Past research for admitted patients found a sensitivity of 33% and PPV of 12% for ESPMv1 using standard alerting thresholds. This has questioned the performance characteristics of the model and highlighted the need for external validation in multiple hospital systems and healthcare settings.^11^^,^^12^

Within Harris Health System in Houston, TX, both Ben Taub and Lyndon B Johnson Hospitals utilize Epic as their EHR. On December 4, 2022, Harris Health implemented the ESPMv1 system-wide. Prior to this production implementation, patient-level data were collected for quality assurance of the ESPMv1’s deployment. This study evaluated previously collected data to externally validate the ESPMv1 with regard to sepsis identification in a county ED setting.

## Materials and methods

### Study design and data source

This retrospective study included all patients aged 18 years or older, evaluated at 2 Harris County EDs from January 1, 2023 to December 31, 2023. This study was approved by the investigator’s institutional review boards with an exception from informed consent due to the retrospective design. The protocol was designed to ensure representation of all race and gender groups in proportions present in the study population without selection bias.

### Sepsis definition

Sepsis was defined using Third International Consensus Definitions for Sepsis and Septic Shock (Sepsis-3) as an increase in 2 points on the Sequential Organ Function Assessment (SOFA) score^13^ in a patient where blood cultures and antibiotics were ordered. The electronic medical record was queried in 15-minute intervals for the data required for SOFA score calculations.^14^^,^^15^ The time of onset of sepsis was recorded as the time when the SOFA score changed by 2 or more points. Only patients presenting to the ED were evaluated during the study timeframe. SOFA scores were calculated at 15-minute intervals to match the ESPMv1 frequency while the patient was in the ED and for the entirety of their hospital stay for subsequent timing analysis.

### ESPM evaluation

The ESPMv1 generated numeric categorization of the patient-level data in 15-minute intervals with the threshold for clinician alerting set at model threshold of 6 as recommended by Epic and utilized in the hospital’s production environment.^16^ We calculated the sensitivity, specificity, and predictive values for the ESPMv1 for patients during the Epic recommended 6-hour time window and also for the entirety of the hospital encounter. The occurrence of sepsis and the relation to the Epic alert was also evaluated for the duration of the hospital encounter. The EPSMv1 produces an alert to physicians and nurses in the form of an interruptive best practice advisory (BPA) alert. A provider must view the patient’s chart and the EPSMv1 must have achieved a threshold of 6 to display the BPA. Statistical analysis was performed using R Statistical Software (v4.1.2; R Core Team 2021; glm package) for multi-level Race variable’s *P*-values and for binomial distribution regressing on the sepsis outcome. Binary variables’ *P*-values were calculated using Microsoft Excel (Redmond, WA). Using Monte Carlo simulation (R v4.1.2), we also generated a random alert as a comparison. The random alert was not shown to physicians but served as a baseline for evaluating predictive outcomes.

## Results

**Figure 1.**
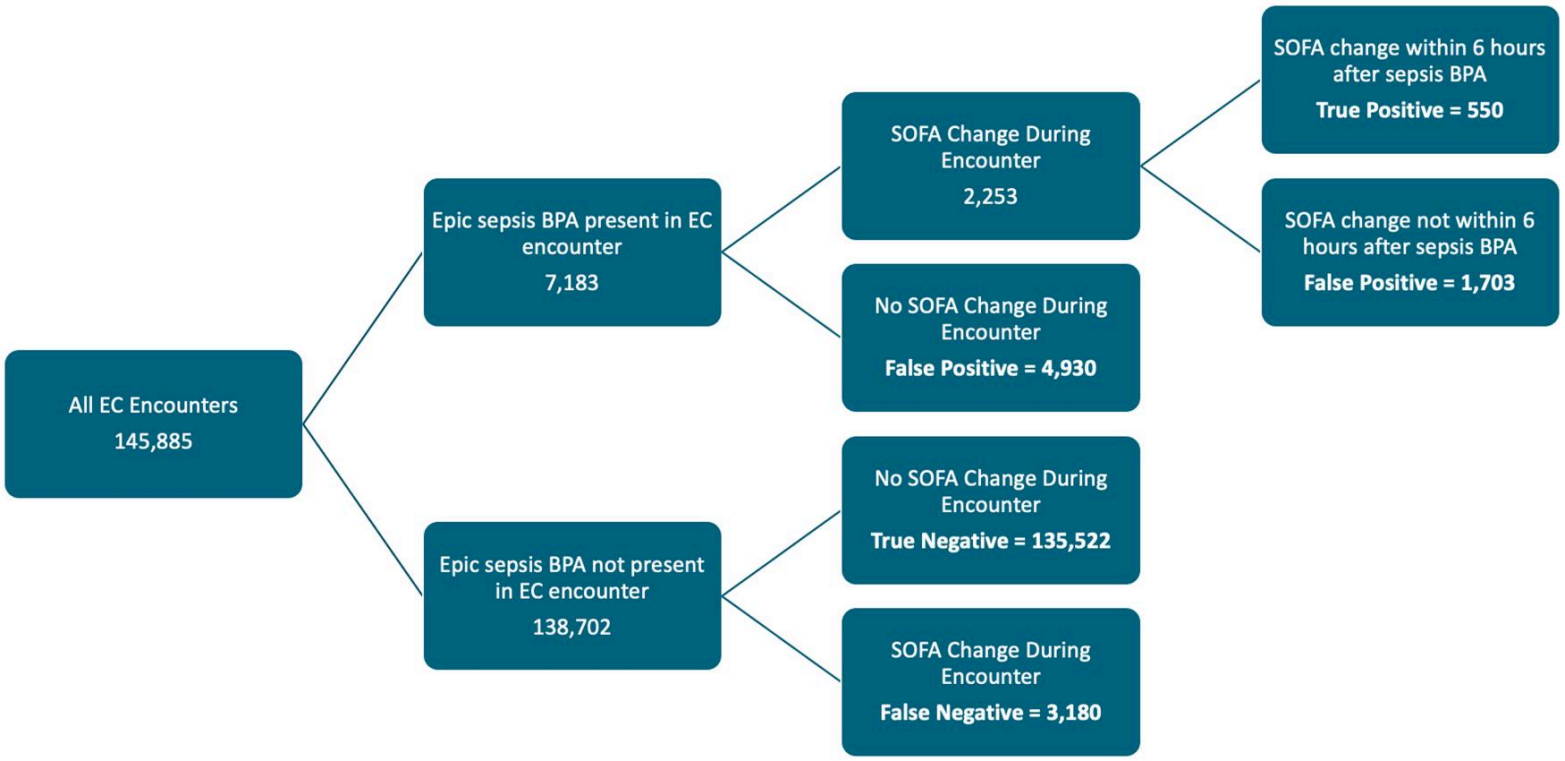
Patient flow diagram.

We evaluated 145 885 patient encounters initiated in the ED across 2 hospitals from January 1, 2023 to December 31, 2023. The ESPMv1 BPA was present in 7183 (5%) encounters and not present in 138 702 (95%) encounters. A total of 5433 (3.7% of all encounters) met the definition for sepsis with 550 (10% of sepsis encounters) occurring within 6 hours after the BPA alert. In 138 702 encounters where the ESPMv1 BPA was not present, 3180 encounters were treated for sepsis (Figure 1).

Within a 6-hour time window of alert firing, we calculated a sensitivity of 14.7%, specificity of 95.3%, PPV of 7.6%, and negative predictive value (NPV) of 97.7% (Table 1). If the window of sepsis prediction was extended to the entirety of the hospital encounter, sensitivity increased to 41.7%, specificity 96.5%, PPV of 31.4%, and NPV of 97.7% (Table 2).

**Table 1.** Confusion matrix for sepsis occurrence within 6 hours of ESPMv1 BPA.

	Sepsis (*n* = 3730)	Not sepsis (*n* = 142 155)	
**ESPMv1 positive**	550	6633	PPV 7.6%
**ESPMv1 negative**	3180	135 522	NPV 97.7%
	Sensitivity 14.7%	Specificity 95.3%	

**Table 2.** Confusion matrix for sepsis occurrence during entirety of hospital encounter.

	Sepsis (*n* = 5433)	Not sepsis (*n* = 140 452)	
**ESPMv1 positive**	2253	4930	PPV 31.4%
**ESPMv1 negative**	3180	135 522	NPV 97.7%
	Sensitivity 41.5%	Specificity 96.5%	

### Comparison to random alerting

The random alert not shown to clinicians generated a 1.27% sensitivity, 95.5% specificity, PPV of 0.41%, and a 98% NPV. Adjustment of the ESPMv1 for randomness yields a new sensitivity of 13.43% within the 6-hour time window.

### Timing analysis

**Figure 2.**
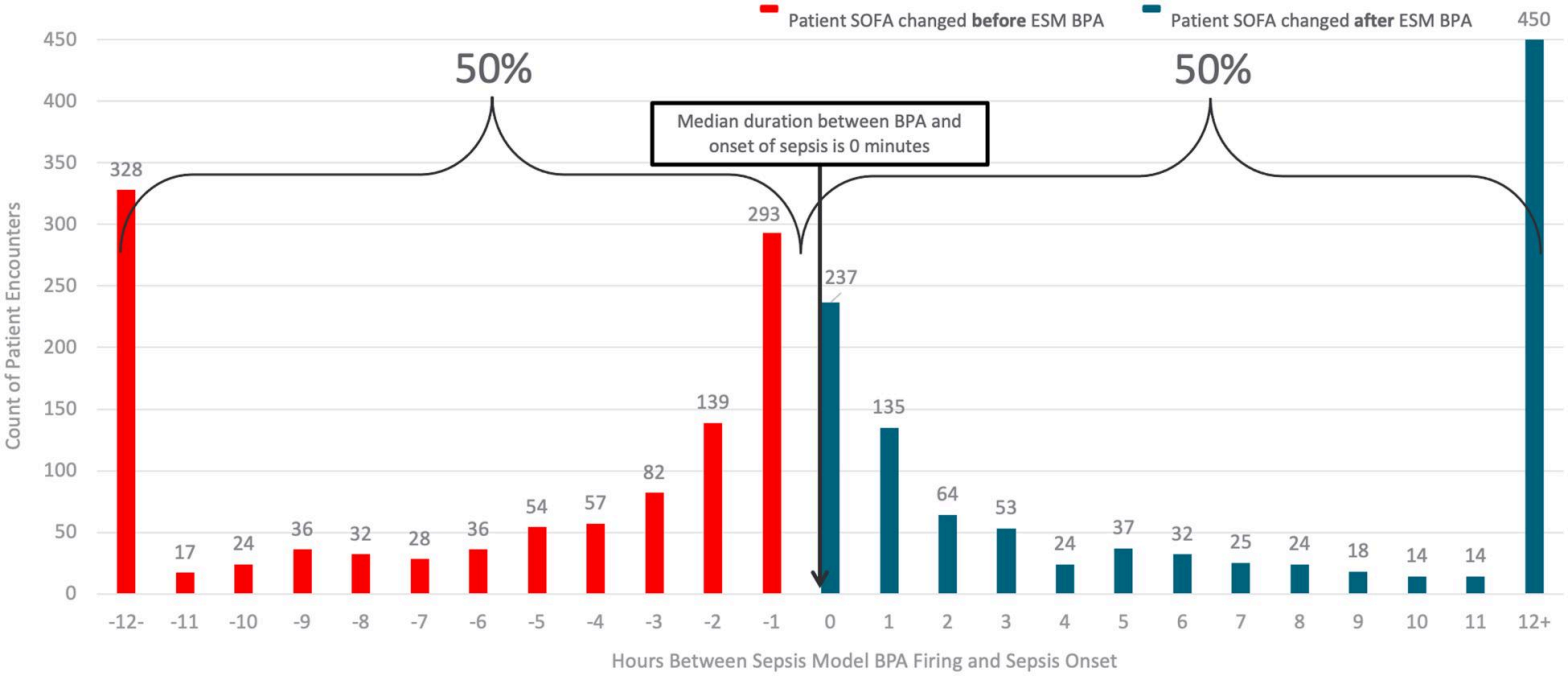
Timing analysis for patients with ESPMv1 BPA alerting and SOFA change.

To evaluate the possibility of alert fatigue and the clinical relevance of sepsis alerts, we analyzed the 2253 patient encounters with a ESPMv1 alert and sepsis present. In 50% (n = 1126) of these encounters, the ESPMv1 alerted after sepsis occurred while 1126 (50%) had an ESPMv1 alert 6 hours or less prior to the occurrence of sepsis. In the encounters where the ESPMv1 alerted prior to sepsis, the median time between the ESPMv1 and sepsis occurring was 0 minutes (80% CI, −6 hours and 42 minutes to 12 hours and 0 minutes) (Figure 2).

## Discussion

Our external validation study of the ESPMv1 demonstrates poor diagnostic characteristics as shown in prior validations^12^ with a PPV of 7.6%. We also report similarly poor sensitivity of 14.7.0% and a specificity of 95.3% for the occurrence of sepsis within 6 hours of alerting the provider. Our validation represents the largest external validation to date of the ESPMv1 in a county hospital ED setting. Our results align with previously published external validations for the ESPMv1^12^ and slightly worse than Epic’s published analysis.^16^ Additionally, when comparing the alert to a random alert, we saw marginally better performance regarding sensitivity and specificity. Due to the rarity of sepsis as a disease, the NPV is mostly attributable to randomness and disease infrequency. With regard to sensitivity, many of the clinicians, as evidenced by the timing analysis, are treating sepsis independent of alerting.

In our population, the ESPMv1 alerted providers with a median lead time of 0 minutes (80% CI, −6 hours and 42 minutes to 12 hours and 0 minutes) and only alerted providers in half of the cases prior to sepsis occurrence. This suggests that the ESPMv1 alert is adding little assistance to physicians identifying sepsis. With clinicians treating sepsis 50% of the time without an alert, pop-ups can only marginally assist in disease identification. There is also an element of alert fatigue^17^ that has been well described with relation to alerts that provide information to physicians when they were already aware of a disease state.^18^ Although it is unknown if sepsis would have been successfully treated without the alert in 50% of cases, it is unlikely given the distribution, to suggest that those cases would have been completely missed.

The ED county population studied in this dataset, represented a predominately Hispanic (59%) and Black (26%) cohort in a lower socioeconomic bracket (Table 3).^19^ This most likely differs significantly from the original derivation population and subsequent studied groups.^11^^,^^16^ In addition, the original EPIC derivation of this model did not utilize Sep-3 as the definition of sepsis and instead utilized lactic acid values for classification.^16^ Prior research has described the racial differences in sepsis outcomes and incidence and hypothesized such differences may have both socioeconomic and genetic factors.^20^^,^^21^ In our dataset, these racial differences most likely contribute to a significant deviation in the alert’s diagnostic characteristics. Although future models will adjust to the changing landscape of sepsis definitions and clinical care, derivation and validation in representative populations will be necessary for all deployed alerting systems.

**Table 3.** Patient characteristics grouped by occurrence of septic for entirety of hospital encounter.

	No sepsis *N* = 140 452 (%)	Sepsis *N* = 5433 (%)	*P*-value^a^
**Sex**			
Female	64 073 (96.8)	2100 (3.2)	<.01
Male	76 379 (95.8)	3333 (4.2)	
**Age (years)**			
18-44	65 009 (97.9)	1375 (2.1)	
45-64	53 005 (95.2)	2673 (4.8)	<.01
65+	22 438 (94.2)	1385 (5.8)	
**Race**			
Asian	1310 (94.7)	74 (5.3)	
Black	42 419 (96.8)	1418 (3.2)	
Hispanic	77 670 (96.0)	3243 (4.0)	.2
White	14 411(96.2)	568 (3.8)	
Other	4624 (97.3)	130 (2.7)	
Hypertension			
Diabetes	31 288 (93.1)	2324 (6.9)	<.01
Hyperlipidemia	33 828 (94.1)	2134 (5.9)	<.01
Chronic kidney disease	1581 (87.7)	222 (12.3)	<.01
Coronary artery bypass graft	3671 (93.0)	276 (7.0)	<.01
Peripheral vascular disease	3427 (89.2)	415 (10.8)	<.01
Stroke	3896 (91.3)	369 (8.7)	<.01

a Statistical tests performed: χ^2^ test of independence.

Predictive models without testing against unguided clinician performance cannot prove superiority or value added to patient care. Since all alerting models seek to augment the performance of clinicians, their baseline level of diagnostic values should exceed that of the physician if they are to improve bedside care. Future research must focus on patient-centered outcomes of sepsis alerting such as overall hospital length of stay, time to antibiotics, and mortality reductions. Alerting thresholds should be evaluated in reference to provider types such as different thresholds for nursing and physician groups.

### Limitations

Our paper is limited by inclusion of Sep-3 definition and the calculation of SOFA scores via the EHR. Although widely accepted as an EHR level definition for abstracting sepsis data, misclassification of patients may have occurred as the exact occurrence of sepsis is unclear and relies upon the change in measurements of end organ dysfunction. Also, the ESPMv1 alert may have influenced provider action by encouraging antibiotic and cultures since the alert was active during the entirety of our dataset. The ordering of antibiotics and cultures may influence the model scoring and influence the time to physician alerting. Our validation was only performed at a single county health system that includes 2 hospital locations. Our dataset was limited to ED patients and to the usage of this version of the predictive model in our production environment. We only evaluated version 1.0 of the ESPM, and future changes to model weighting and thresholds may alter the alert’s diagnostic characteristics. We were unable to assess the clinical outcomes from the timing of this alert.

## Conclusions

Our study confirms the need for hospital-level validation of EHR-based prediction models. The ESPMv1 alerting fails to achieve meaningful sensitivity and a random alert achieves similar specificity and NPVs. Such a poorly functioning alert for a time critical disease has wider physician-level implications regarding alert fatigue and clinical outcomes.

## Data Availability

The data underlying this article were accessed from Harris Health System’s electronic medical records from January 1, 2023 to December 31, 2023. The derived data generated in this research will be shared on reasonable request to the corresponding author subject to the approval by the associated medical institutions.
